# HPV Vaccination during the COVID-19 Pandemic in Italy: Opportunity Loss or Incremental Cost

**DOI:** 10.3390/vaccines10071133

**Published:** 2022-07-16

**Authors:** Francesco Saverio Mennini, Andrea Silenzi, Andrea Marcellusi, Michele Conversano, Andrea Siddu, Giovanni Rezza

**Affiliations:** 1 Economic Evaluation and HTA (EEHTA CEIS), Department of Economics and Finance, Faculty of Economics, University of Rome “Tor Vergata”, Via Columbia 2, 00133 Rome, Italy; mennini@uniroma2.it; 2 Institute for Leadership and Management in Health, Kingston University, River House, 53–57 High Street, Kingston upon Thames, London Surrey KT1 1LQ, UK; 3 General Directorate for Health Prevention, Ministry of Health, Viale Giorgio Ribotta 5, 00144 Rome, Italy; a.siddu@sanita.it (A.S.); g.rezza@sanita.it (G.R.); 4 Economic Evaluation and HTA (EEHTA CEIS), Faculty of Economics, University of Rome “Tor Vergata”, Via Columbia 2, 00133 Rome, Italy; andrea.marcellusi@uniroma2.it; 5 Department of Prevention, Local Health Authority of Taranto, Via Diego Peluso 117, 74121 Taranto, Italy; michele.conversano@asl.taranto.it

**Keywords:** HPV, HTA, incremental cost, RWD, COVID-19, vaccination strategies

## Abstract

Objectives: Italy was the first European country to introduce universal vaccination of adolescents, for both males and females, against Human Papilloma Virus (HPV) starting in 2017 with the NIP 2017–2019′s release. However, vaccine coverage rates (VCRs) among adolescents have shown a precarious take-off since the NIP’s release, and this situation worsened due to the impact of the COVID-19 pandemic in 2020. The aim of this work is to estimate the epidemiological and economic impact of drops in VCRs due to the pandemic on those generations that missed the vaccination appointment and to discuss alternative scenarios in light of the national data. Methods: Through an analysis of the official ministerial HPV vaccination reports, a model was developed to estimate the number of 12-year-old males and females who were not vaccinated against HPV during the period 2017–2021. Based on previously published models that estimate the incidence and the economic impact of HPV-related diseases in Italy, a new model was developed to estimate the impact of the aggregated HPV VCRs achieved in Italy between 2017 and 2021. Results: Overall, in 2021, 723,375 girls and 1,011,906 boys born between 2005 and 2009 were not vaccinated against HPV in Italy (42% and 52% of these cohorts, respectively). As compared with the 95% target provided by the Italian NIP, between 505,000 and 634,000 girls will not be protected against a large number of HPV-related diseases. For boys, the number of the unvaccinated population compared to the applicable target is over 615,000 in the ‘best case scenario’ and over 749,000 in the ‘worst case scenario’. Overall, between 1.1 and 1.3 million young adolescents born between 2005 and 2009 will not be protected against HPV-related diseases over their lifetime with expected lifetime costs of non-vaccination that will be over EUR 905 million. If the 95% optimal VCRs were achieved, the model estimates a cost reduction equal to EUR 529 million, the net of the costs incurred to implement the vaccination program. Conclusion: Suboptimal vaccination coverage represents a missed opportunity, not only because of the increased burden of HPV-related diseases, but also in terms of economic loss. Thus, reaching national HPV immunization goals is a public health priority.

## 1. Introduction

In 2008, Prof. H. Zur Hausen received the Nobel Prize for discovering the cancerogenic role of HPV, which he initially discovered in 1976. Following that, in 1989, Prof. Ian Frazer discovered the HPV vaccine that later in 2006 became available globally [1]. After the introduction of the vaccine, the clinical information concerning HPV changed considerably. At the time of its introduction, the HPV vaccine was mostly used for the prevention of cervical cancer in women. A few years later, the role of HPV as a causative agent of gender-neutral cancers was proved, namely anal, oral and head and neck cancers [2]. In 2015, a new version of the vaccine, active on a larger number of HPV strains that cause malignancies [3,4], was introduced.

After many studies showed both the cost effectiveness and the efficiency of HPV vaccination in women [5,6], the BEST II study evaluated the cost-effectiveness of universal vaccination compared with selective vaccination of 12-year-old girls and the economic impact of immunization on various HPV-induced diseases [7]. In this paper, a dynamic Bayesian Markov model was developed to investigate the transmission of HPV in cohorts of females and males. As a result, gender-neutral HPV vaccination was found to be a cost-effective alternative when compared with either cervical cancer screening or female-only vaccination. Based on this new evidence, the Italian government was the first among the G8 Countries to extend the HPV national immunization program to 12-year-old boys (2017). This gender-neutral strategy is sanctioned by the 2017–2019 National Immunization Program (NIP) update, which extends vaccination to males and defines increasing coverage targets for both sexes to reach the 95% coverage target expected for 2019 and keep it stable for the years to come [8].

The achievement of high coverage in the population, as well as for other vaccinations, guarantees indirect protection (herd immunity) and, consequently, represents an important public health goal. In Italy, the data on vaccination coverage are provided by the regions to the Ministry of Health and are published annually. The ministerial reports, albeit with some data gaps, represent the official source of monitoring the coverage objectives envisaged by the 2017–2019 NIP currently in force.

To a generalized picture of precarious take-off of this vaccination, especially in males, in 2020, the impact of the COVID-19 pandemic was added, which lasted into 2021, as a result of the commitment at the forefront of the vaccination centers in the influenza and COVID-19 vaccination campaigns. In July 2020, the Ministry of Health summarized the results of an investigation conducted on the vaccination centers in the aftermath of the outbreak of the pandemic. The survey revealed that 97 local health authorities (LHAs) witnessed a decrease in vaccinations; in 68% of cases, it was adolescents’ vaccinations that had suffered the greatest delay due to the pandemic [9]. Adolescents also experienced the closure of schools, limitation to sociability and a reduction in opportunities for contact with the privileged points of information and the booking of vaccinations.

This study, in light of the above, aims to estimate the epidemiological and economic impact of the drop in vaccine coverage rates (VCRs) due to the pandemic on those generations that missed the vaccination [10], and, in addition, it aims to discuss different estimates of the impact of the pandemic and possible organizational responses.

## 2. Methods

Two previously published models in Italy estimated the impact of HPV-related diseases on the Italian population regarding health conditions and death risk [11,12]. These works evaluate the effect of vaccination over a single cohort of Italian males and females. Starting from these simulations, a new model was developed to estimate the impact of the aggregated HPV VCRs achieved in Italy between 2017 and 2021.

The analysis develops according to three different steps:

1. Vaccine coverage rates’ (VCRs) scenarios definition: by extracting the VCRs recorded in the official ministerial HPV vaccination reports for the cohorts of girls and boys born between 2005 and 2008 (eligible on their 12th year of age and therefore vaccinated between 2017 and 2020), two scenarios were developed:
  - Achieved VCRs: in this scenario, we considered the actual vaccination coverage rate recorded in Italy for cohorts 2005–2008 reported from the official ministerial HPV vaccination reports, and we made assumptions of VCRs achieved in the 2009 cohort, whose VCRs are not publicly available at the moment of this publication [10].
  - Target coverage rate: in this second scenario, the optimal vaccine target defined by the Italian NIP 2017–2019 was considered [8] (Table 1).
2. Model adaptation for gender, coverage rate and efficacy: the previously developed model, considered the starting point for this analysis [13,14], includes only bivalent and quadrivalent vaccination in their simulations. However, in Italy, in 2017, a nine-valent vaccination program was adopted. Vaccine-specific efficacy data were updated and adapted to the coverage rates considered in the scenario analysis, and a specific bibliography was considered for HPV9-related disease rates (Table 2). Event rates were also updated to consider the most recent hospital admissions in Italy as identified through administrative archives.
3. Model simulation and economic effects: the model was performed considering the assumed scenarios and absolute differences were calculated for HPV-related events, direct and indirect costs.

### 2.1. Scenarios Definition

Every year, the Italian Ministry of Health publishes national and regional coverage data for vaccination against HPV in the female and male populations [10]. The model considered the rates of a complete vaccination cycle (two doses) applied to the 11-year-old resident population on 1 January of each year (Table 1) [15]. The same approach was considered for the target scenario, which were reported according to the Italian NIP and were meant to increase gradually for boys, reaching the 95% target from the 2007 male cohort onwards [8].

Given that the last ministerial report refers to VCRs performed in the 2008 cohort during the pandemic year (2020) and that this cohort might have undergone a catch-up in 2021, the model will make some assumptions regarding the VCRs in these two cohorts (scenario analysis). For the purpose of the simulation and to account for two different scenarios of COVID-19′s impact on VCRs on the most affected cohorts, the model defines a ‘worst case scenario’ and a ‘best case scenario’ as follows:

- ‘Worst case scenario’: Coverage rates for males and females vaccinated in the years 2020 and 2021 (birth cohorts 2008 and 2009) remain the same as for those registered in 2020 (30.3% and 24.2% for female and male, respectively) [10]. No catch-up for the 2008 birth cohort was assumed;
- ‘Best case scenario’: in this scenario, the model assumes a national coverage rate for the two cohorts equal to the maximum level registered in the Italian regions in 2020 (53.4% in Tuscany registered for females and 46.9% registered in Emilia Romagna for males) [10].

**Table 2:** Input parameters.

	Preventable Fraction of Desease		
Parameter	Base-Case Value	Min–Max	Source
**Vaccine Efficacy data (Reduction rate)**			
CIN2+	97.1%	83.5–99.9	RCP Gardasil 9^®^
Cervical cancer	97.4%	85–99.9	RCP Gardasil 9^®^
NIV2+	100%	55.5–100	RCP Gardasil 9^®^
Vaginal cancer	97.4%	85–99.9	RCP Gardasil 9^®^
Vulvar cancer	97.4%	85–99.9	RCP Gardasil 9^®^
Penis cancer	100%	52–100	RCP Gardasil^®^
Anus cancer	74.9%	8.8–95.4	RCP Gardasil 9^®^
Oropharyngeal cancer	77.5%	39.6–93.3	RCP Gardasil 9^®^
Genital condylomas	99%	96.2–99.9	RCP Gardasil 9^®^
Recurrent respiratory papillomatosis	90.7%	81–100%	Assumption
**Annual outpatient cost**			
CIN2+	EUR 498	+/−20%	[14]
NIV2+, CIS	EUR 498	+/−20%	[14]
Cervical Cancer	EUR 202	+/−20%	[14]
Vulvar + vaginal cancer	EUR 202	+/−20%	[14]
Penis cancer	EUR 202	+/−20%	[14]
Anus cancer	EUR 279	+/−20%	[14]
Oropharyngeal cancer	EUR 202	+/−20%	[14]
Genital condylomas	EUR 704	+/−20%	[14]
Recurrent respiratory papillomatosis	EUR 202	+/−20%	[14]
**Annual indirect cost**			
CIN2-3	EUR 8333	+/−20%	[16]
NIV2+, CIS	EUR 8333	+/−20%	[16]
Cervical Cancer	EUR 9130	+/−20%	[16]
Vulvar + vaginal cancer	EUR 9122	+/−20%	[16]
Penis cancer	EUR 9131	+/−20%	[16]
Anus cancer	EUR 9128	+/−20%	[16]
Oropharyngeal cancer	EUR 9310	+/−20%	[16]
Genital condylomas	-	-	[16]
Recurrent respiratory papillomatosis	EUR 9310	+/−20%	[16]
**HPV9 related disease**			
CIN2+	82.3%	+/−20%	[17,18]
Cervical Cancer	89.1%	+/−20%	[17,18]
NIV2+	94.4%	+/−20%	[17,18]
Vaginal cancer	67.9%	+/−20%	[17,18]
Vulvar cancer	45.3%	+/−20%	[17,18]
Penis cancer	46.3%	+/−20%	[17,18]
Anus cancer	94.4%	+/−20%	[17,18]
Oropharyngeal cancer	23.4%	+/−20%	[17,18]
Genital condylomas	90%	+/−20%	[17,18]
Recurrent respiratory papillomatosis	100%	+/−20%	[17,18]

### 2.2. Model Adaptation and Input Parameters

All hospital admissions were identified through administrative archives, according to the International Classification of Diseases (ICD-9 CM). Information related to the hospital discharges of all accredited public and private hospitals, both for ordinary and day care regimes, was taken into account.

We included hospital admissions related to resident patients presenting with one of the ICD-9-CM codes as a primary or secondary diagnosis for: genital warts (GW): ‘condyloma acuminatum’ (078.11); ‘cervical intraepithelial neoplasia (CIN)’ (067.2, 067.32, 067.33 and 067.39); ‘anal cancers’ (AC) (154.2–154.8); ‘oropharyngeal cancers (OC): ‘oropharyngeal cancer’ (146.0–146.9) and ‘head, face and neck cancers’ (171.0); and genital cancers (GC): ‘penis cancer’ (187.1–187.9) and ‘cervical cancer’ (180.0–180.9) [13,14]. Data were stratified by birth years, age of hospitalization and ICD-9-CM group.

### 2.3. Statistical and Sensitivity Analysis

A deterministic analysis was performed considering the estimated age-relative risk of event, the estimated efficacy and the direct and indirect costs applied to the estimated live population of the cohort at the same age. Real-world data were applied to the unvaccinated cohorts, which considers the overall hospitalization rates for each disease, as already done in the Marcellusi, Mennini et al. study [12]. For the vaccinated cohorts, a disease incidence reduction rate was applied, which considers the vaccine efficacy available in the literature and the fraction of each disease attributable to the 9 HPV genotypes included in the nono-valent vaccine available for the years considered in the analysis in Table 2 [7,11]. The number of hospitalizations by age was multiplied by the average hospitalization costs for each age estimated by the diagnosed related groups (DRGs) tariff, the estimated lifetime outpatient costs and social security benefit costs (Table 2). Social security benefit costs took disability benefits (DBs) and incapacity pensions (IPs), estimated from the Mennini et al. study [16], into consideration and applied them to the 15% of CIN patients and 90% of all other disease diagnosed patients (Table 2). Finally, cost and risk reduction were estimated as the absolute and percentage differences between the base-case and target coverage rate scenarios.

The uncertainty associated with the model’s outcomes was estimated through a deterministic sensitivity analysis (DSA). In the DSA, each sensible parameter of the model was subject to a variation derived from the literature (CI of vaccine efficacy data) or from an arbitrary variation [19], as reported in Table 2. The model results derived from each variation were compared to the value of the base case and represented by a tornado diagram.

## 3. Results

**Figure 1:**
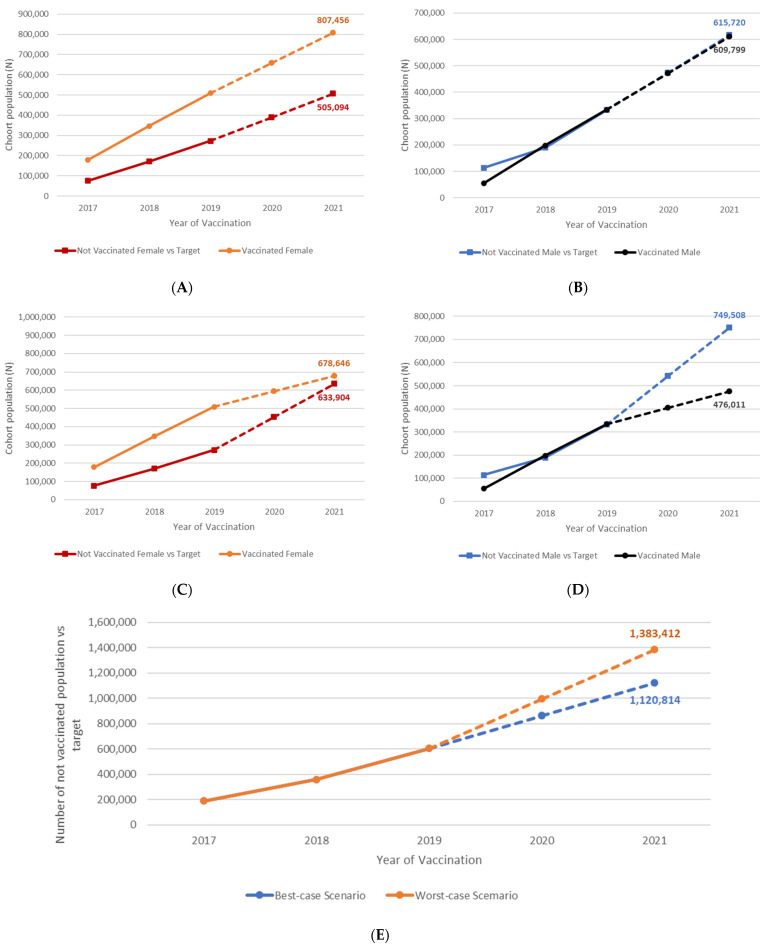
Unvaccinated population according to two scenarios vs. NIP optimal target. (**A**) Pandemic ‘best case scenario’ for coverage rates between 2020 and 2021, female; (**B**) Pandemic ‘best case scenario’ for coverage rates between 2020 and 2021, male; (**C**) Pandemic ‘worst case scenario’ for coverage rates between 2020 and 2021, female; (**D**) Pandemic ‘worst case scenario’ for coverage rates between 2020 and 2021, male; (**E**) Unvaccinated population according to two scenarios vs. NIP optimal target.

In 2021, 723,375 girls and 1,011,906 boys born between 2005 and 2009 were not vaccinated against HPV in Italy (42% and 52% of these cohorts, respectively). As compared with the 95% target provided by the Italian NIP, between 505,000 and 634,000 girls will not be protected against a large number of HPV-related diseases. For boys, the number of the unvaccinated population compared to the applicable target is over 615,000 in the ‘best case scenario’ and over 749,000 in the ‘worst case scenario’ (Figure 1).

Overall, between 1.1 and 1.3 million young adolescents born between 2005 and 2009 will not be protected against HPV-related diseases during their lifetime, and the expected lifetime costs of non-vaccination will be over EUR 905 million. If the 95% optimal VCRs were achieved, the model estimates a cost reduction equal to EUR 529 million, the net of the costs incurred to implement the vaccination program.

**Figure 2:**
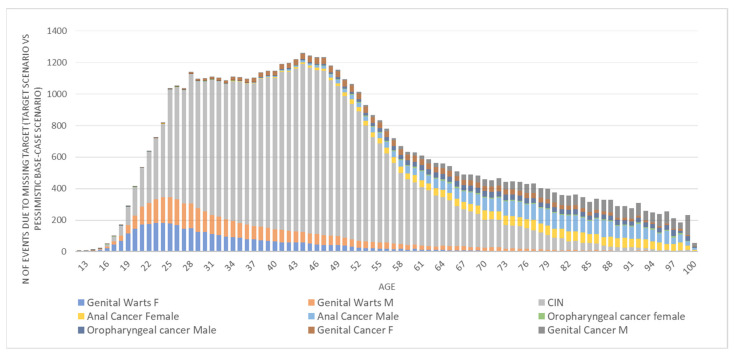
HPV-related diseases developed by the 2005–2009 cohorts in the ‘worst case scenario’, which are avertable upon achievement of the optimal target for VCRs.

Figure 2 shows the forecasted number of events, by HPV-related conditions, attributed to the suboptimal VCRs vs. the NIP’s immunization targets over the years (age of the population is considered). The model estimates that over 1200 events annually could be averted if the target for vaccination was reached. GWs and CIN cases represent the most common events in the younger ages of the population, while cancers are the most frequent, though less incident, in the older ages.

The model also estimated the lifetime cost associated with the five simulated cohorts by considering three different scenarios. Without the HPV immunization program (scenario (a), no vaccination), the expected lifetime costs would be over EUR 905 million (54% due to hospitalization, 38% indirect and 8% outpatients’ costs). Assuming the VCRs ‘worst scenario’ due to the pandemic were true, the sub-optimal coverage rates performed between 2017 and 2021 would lead to a model estimate of the cost reduction equal to EUR 260 million (−28% vs. no vaccination scenario), with a significant economic burden for the NHS of the EUR 644,618,178 of HPV-related diseases. Finally, if VCRs scored up to the optimal NIP targets for the selected cohorts (60%, 95% according to sex and year of eligibility), an additional EUR 269 million could be saved (−58% vs. no vaccination scenario). To synthesize, protecting adolescents born between 2005 and 2009 by reaching the NIP’s optimal HPV immunization rates would potentially avoid up to EUR 529,6 million in the lifetime horizon as compared to ‘no vaccination’. The COVID-19 pandemic is likely to have compromised the VCRs of the 2008 and 2009 cohorts as modelled in our ‘worst case scenario’. In this scenario, which also account for sub-optimal VCRs in the 2005–2007 cohorts, the total economic and social burden of HPV-related conditions will remain significantly high (Table 3).

**Figure 3:**
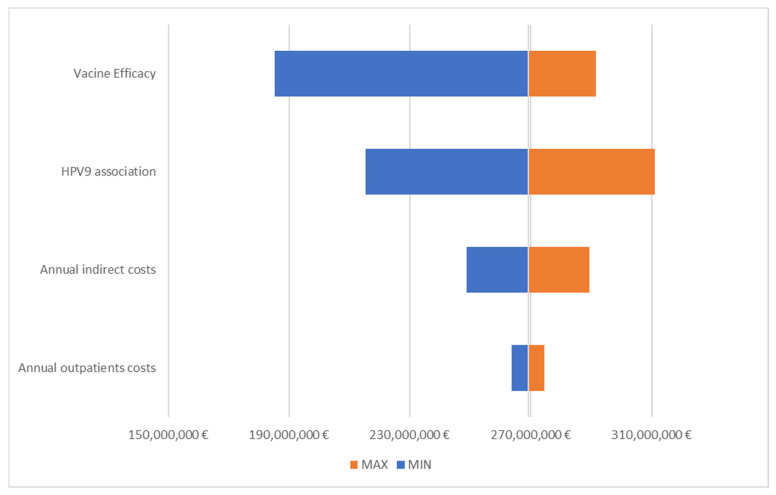
Tornado diagram for incremental savings at target VCRs.

Figure 3 shows that the model was sensitive to the vaccine efficacy and association of HPV9 diseases. In all tested scenarios, the incremental savings at the target VCRs was between EUR 185 (−31% vs. the base case analysis) and EUR 311 (+16% vs. the base-case analysis) million.

## 4. Discussion

This analysis shows, in an intuitive and accurate way, the epidemiological and economic impact of the ‘lost generations’ to anti-HPV vaccination due to sub-optimal historic VCRs and to the likely impact of the pandemic on the 2020 and 2021 immunization campaigns [20]. This is an extremely negative impact that may begin to have important consequences, in terms of the burden of disease, on these generations when they become young adults of a productive age.

In light of these results, it becomes important to stress HPV immunization as a public health priority and to identify organizational solutions capable of promoting adolescent-friendly prevention, vaccine confidence and a full recovery of coverage after the pandemic emergency [21,22,23].

The PNPV 2017–2019, which is still in force as it was extended for the COVID-19 pandemic emergency, defines a flowchart addressed to achieve high coverage through a repeated and carefully monitored ‘invite and remind’ system and presents the operating procedures to promote the active and free offer of vaccinations [8]. Since HPV vaccination is not mandatory—it is only recommended—effective organizational models to offer vaccination and the ability to engage the target population and their parents have become strategic [24,25,26].

A consensus conference held in 2019 issued a series of recommendations for implementing HPV vaccination that were recognized to be highly recommended both by the international literature and by the opinions of Italian experts, based on the most effective recognized strategies [27].

The included recommendations were:

(i) Sending reminders to parents of 11–12-year-olds a few days before the agreed upon vaccination appointment following the active call from the vaccination program and in the case of a no-show at an agreed appointment;

(ii) The implementation of reminder strategies aimed at health professionals to remind them to propose vaccination during a visit to a citizen who has not been previously vaccinated, but for whom vaccination is planned. In particular, it is recommended to activate reminder strategies aimed at the pediatrician of free choice (PLS), the heads of the vaccination clinic and general practitioners (GPs);

(iii) The provision of informational/educational strategies aimed at vaccination targets and/or their parents are weakly recommended since informational/educational actions for the population are generally ineffective in increasing compliance with vaccination and may encounter economic sustainability problems;

(iv) The implementation of reminders/informational activities should, on the other hand, be based mainly on the direct trust relationship between the health worker and the vaccination target/parents of the target or be implemented at the school level.

The consensus identified, while acknowledging the difficult implementation of the strategy, that secondary school is the ideal venue for information, education and vaccination opportunities.

The implementation of strategies that provide, among other interventions, the carrying out of vaccination in the school setting, have proved effective in various contexts, despite encountering organizational difficulties at the local level.

Given the situation of poor coverage and the real risk of missing the HPV immunization for more than half of the boys and girls born after 2004, it seems speculative to stop at the evidence of the literature and their transferability to the Italian context: evaluating alternative vaccination and information settings become an imperative in this circumstance [28]. Countries where vaccination against HPV is school-based (Sweden, Australia) showed high coverage rates in the primary cohort and allowed the carrying out of multi-court catch-up campaigns for children up to 18 years of age [29,30,31].

The COVID-19 pandemic may leave an indelible mark on the reproductive health of millions of adolescents; however, the response to this same emergency has shown us rare examples of resilient organizational systems, and it is hoped that this moment will be also an opportunity to promote real organizational innovation in organizational models for the promotion of vaccine prevention [32,33,34].

## 5. Conclusions

Looking closely at the results of this analysis, it emerges strongly that, overall, between 1.1 and 1.3 million of young adolescents born between 2005 and 2009 will not be protected against HPV-related diseases during their lifetime, with an expected lifetime cost of non-vaccination that will be over EUR 905 million. If the 95% optimal VCRs were achieved, the model estimates a cost reduction equal to EUR 529 million, the net of the costs incurred to implement the vaccination program.

The results of recent studies [35,36] highlight how the inclusion of additional HPV types in the vaccine offers great potential to expand protection against HPV infection and the associated disease burden. However, most importantly, the impact of reducing the global burden caused HPV-related cancer depends on vaccine uptake and coverage, as well as the availability and, finally, its affordability from the perspective of the payer.

Consequently, this analysis highlights and demonstrates how suboptimal vaccination coverage represents a missed opportunity, not only because of the increased burden of HPV-related diseases, but also in terms of economic loss. Thus, reaching national HPV immunization goals is a public health priority.

### Strength and Limitation

The study presents some limitations and some strengths. The lifetime projection model has a high level of uncertainty, though the sensitivity analysis showed important advantages. Again, the model is based on assumptions for 2020–2021 coverage, and yet a scenario analysis was conducted. The study, in light of the highlighted results and in terms of both the economic and epidemiological benefits, is a valuable tool for decision makers to be able to make an informed decision regarding vaccination policies in Italy.

## Figures and Tables

**Table 1:** VCRs rate by scenario.

	Gender	Cohort Vaccinated between 2017–2021				
		2005	2006	2007	2008	2009 *
Resident Population per cohort at 11 years [9]	Female	266,893	277,302	278,826	280,109	278,502
	Male	281,034	291,966	293,260	294,204	294,561
Base-case scenario [8]	Female	66.6%	60.9%	58.3%	30.3%	30.3%
	Male	19.6%	49.0%	46.2%	24.2%	24.2%
Target Scenario [5]	Female	95.0%	95.0%	95.0%	95.0%	95.0%
	Male	60.0%	75.0%	95.0%	95.0%	95.0%

* Estimated considering the same coverage rate as that of the 2008 cohort.

**Table 3:** Direct and indirect lifetime costs associated with different VCRs (aggregate birth cohort 2005–2009).

	(a) No Vaccination	(b) Worst Case Scenario	(c) Target VCRs	Savings Due to Worst Case VCRs (a–b)	Incremental Savings at Target VCRs (c–b)
Hospitalization	EUR 483,668,215	EUR 350,486,959	EUR 209,227,045	EUR 133,181,256	EUR 141,259,914
Outpatient	EUR 75,084,822	EUR 48,368,514	EUR 21,819,584	EUR 26,716,308	EUR 26,548,930
Social Security System	EUR 346,378,005	EUR 245,762,705	EUR 144,493,618	EUR 100,615,300	EUR 101,269,087
Total costs	EUR 905,131,042	EUR 644,618,178	EUR 375,540,247	EUR 260,512,863	EUR 269,077,931

## Data Availability

Publicly available datasets were analyzed in this study. These data can be found here: https://www.salute.gov.it/portale/news/p3_2_1_1_1.jsp?lingua=italiano&menu=notizie&p=dalministero&id=5666 (accessed on 30 January 2022).

## References

[1] Zur Hausen H. (2008). Papillomaviruses to vaccination and beyond. Biochemistry.

[2] Zur Hausen H. (2009). Papillomaviruses in the causation of human cancers—A brief historical account. Virology.

[3] Van Damme P., Olsson S.E., Block S., Castellsague X., Gray G.E., Herrera T., Huang L.-M., Kim D.S., Pitisuttithum P., Chen J. (2015). Immunogenicity and Safety of a 9-Valent HPV Vaccine. Pediatrics.

[4] Castellsagué X., Giuliano A.R., Goldstone S., Guevara A., Mogensen O., Palefsky J.M., Group T., Shields C., Liu K., Maansson R. (2015). Immunogenicity and safety of the 9-valent HPV vaccine in men. Vaccine.

[5] Zhang J., Qin Z., Lou C., Huang J., Xiong J. (2021). The efficacy of vaccination to prevent human papilloma viruses infection at anal and oral: A systematic review and meta-analysis. Public Health.

[6] Mennini F.S., Baio G., Montagano G., Cauzillo G., Locuratolo G., Becce G., Gitto L., Marcellusi A., Zweifel P., Capone A. (2012). Governance of preventive Health Intervention and on time Verification of its Efficiency: The GIOVE Study. BMJ Open.

[7] Haeussler K., Marcellusi A., Mennini F.S., Favato G., Picardo M., Garganese G., Bononi M., Costa S., Scambia G., Zweifel P. (2015). Cost-Effectiveness Analysis of Universal Human Papillomavirus Vaccination Using a Dynamic Bayesian Methodology: The BEST II Study. Value Health.

[8] Italian Ministry of Health National Immunization Program (NIP) 2017–2019. https://www.salute.gov.it/imgs/C_17_pubblicazioni_2571_allegato.pdf..

[9] Italian Ministry of Health Impact of COVID-19 Emergency on Vaccination Activities: Analysis of the Phenomenon and Operational Recommendations. https://www.trovanorme.salute.gov.it/norme/renderNormsanPdf?anno=2020&codLeg=75346&parte=1%20&serie=null.

[10] Italian Ministry of Health Vaccinazione Contro il Papilloma Virus (HPV)—Coperture Vaccinali. https://www.salute.gov.it/portale/documentazione/p6_2_8_3_1.jsp?lingua=italiano&id=27.

[11] Marcellusi A. (2017). Impact of HPV vaccination: Health gains in the Italian female population. Popul. Health Metr..

[12] Marcellusi A., Mennini F.S., Sciattella P., Favato G. (2021). Human papillomavirus in Italy: Retrospective cohort analysis and preliminary vaccination effect from real-world data. Eur. J. Health Econ..

[13] Mennini F.S., Fabiano G., Marcellusi A., Sciattella P., Saia M., Cocchio S., Baldo V. (2018). Burden of Disease of Human Papillomavirus (HPV): Hospitalizations in the Marche and Veneto Regions. An observational study. Clin. Drug Investig..

[14] Mennini F.S., Fabiano G., Favato G., Sciattella P., Bonanni P., Pinto C., Marcellusi A. (2019). Economic burden of HPV9-related diseases: A real-world cost analysis from Italy. Eur. J. Health Econ..

[15] National Institute of Statistics (ISTAT) Resident Population on 1st January. http://demo.istat.it/2022.

[16] Mennini F.S., Nardone C., Gazzillo S., Fabiano G., Migliorini R., Trabucco Aurilio M., Marcellusi A. (2022). HPV9-related diseases: The economic burden of disability benefits and incapacity pensions in Italy. PLoS ONE.

[17] Hartwig S., Lacau St Guily J., Dominiak-Felden G., Alemany L., de Sanjosé S. (2017). Estimation of the overall burden of cancers, precancerous lesions, and genital warts attributable to 9-valent HPV vaccine types in women and men in Europe. Infect. Agent Cancer.

[18] de Martel C., Georges D., Bray F., Ferlay J., Clifford G.M. (2020). Global burden of cancer attributable to infections in 2018: A worldwide incidence analysis. Lancet Glob. Health.

[19] Briggs A.H., Claxton K., Sculpher M.J. (2006). Decision modelling for health economic evaluation. Oxford Handbooks in Health Economic Evaluation, 237.

[20] Gualano M.R., Thomas R., Stillo M., Valentina Mussa M., Quattrocolo F., Borraccino A., Zotti C. (2019). What is the most useful tool in HPV vaccine promotion? Results from an experimental study. Hum. Vaccin. Immunother..

[21] Vorsters A., Bosch F.X., Poljak M., Waheed D.-E.-N., Stanley M., Garland S.M., HPV Prevention and Control Board and the International Papillomavirus Society (IPVS) (2022). HPV prevention and control—The way forward. Prev. Med..

[22] Aninye I.O., Berry-Lawhorn J.M., Blumenthal P., Felder T., Jay N., Merrill J., Messman J.B., Nielsen S., Perkins R., Rowen T. (2021). Gaps and Opportunities to Improve Prevention of Human Papillomavirus-Related Cancers. J. Womens Health.

[23] Sabbatucci M., Odone A., Signorelli C., Siddu A., Silenzi A., Maraglino F.P., Rezza G. (2022). Childhood Immunisation Coverage during the COVID-19 Epidemic in Italy. Vaccines.

[24] Silenzi A., Poscia A., Gualano M.R., Parente P., Kheiraoui F., Favaretti C., Siliquini R., Ricciardi W. (2017). An effective clinical leadership to strenghten the immunization policies in Italy. Ig. Sanita Pubbl..

[25] Cocchio S., Bertoncello C., Baldovin T., Fonzo M., Bennici S.E., Buja E., Majori S., Baldo V. (2020). Awareness of HPV and drivers of HPV vaccine uptake among university students: A quantitative, cross-sectional study. Health Soc. Care Community.

[26] Brunelli L., Bravo G., Romanese F., Righini M., Lesa L., Odorico A.D., Bastiani E., Pascut S., Miceli S., Brusaferro S. (2021). Beliefs about HPV vaccination and awareness of vaccination status: Gender differences among Northern Italy adolescents. Prev. Med. Rep..

[27] Acampora A., Grossi A., Colamesta V., Barbara A., Causio A., Calabrò G.E., Boccia S., Cicchetti A., de Waure C. (2019). Strategies to achieve HPV-related disease control in Italy: Results from an integrative approach. Epidemiol. Biostat. Public Health.

[28] Trucchi C., Costantino C., Restivo V., Bertoncello C., Fortunato F., Tafuri S., Amicizia D., Martinelli D., Paganino C., Piazza M.F. (2019). Immunization Campaigns and Strategies against Human Papillomavirus in Italy: The Results of a Survey to Regional and Local Health Units Representatives. Biomed. Res. Int..

[29] Grandal M., Rosenblad A., Stenhammar C., Tydén T., Westerling R., Larsson M., Oscarsson M., Andrae B., Dalianis T., Nevéus T. (2016). School-based intervention for the prevention of HPV among adolescents: A cluster randomised controlled study. BMJ Open.

[30] Regan D.G., Hocking J.S. (2015). Greatest effect of HPV vaccination from school-based programmes. Lancet Infect. Dis..

[31] Canfell K., Egger S., Velentzis L.S., Brown J.D., O’Connell D.L., Banks E., Sitas F. (2015). Factors related to vaccine uptake by young adult women in the catch-up phase of the National HPV Vaccination Program in Australia: Results from an observational study. Vaccine.

[32] Gilkey M.B., Bednarczyk R.A., Gerend M.A., Kornides M.L., Perkins R.B., Saslow D., Sienko J., Zimet G.D., Brewer N.T. (2020). Getting Human Papillomavirus Vaccination back on track: Protecting our national investment in Human Papillomavirus vaccination in the COVID-19 Era. J. Adolesc. Health.

[33] Ryan G., Askelson N.M., Miotto M.B., Goulding M., Rosal M.C., Pbert L., Lemon S.C. (2022). Lessons learned from Human Papillomavirus vaccination to increase uptake of adolescent COVID-19 vaccination. J. Adolesc. Health.

[34] Signorelli C., Odone A., Gianfredi V., Capraro M., Kacerik E., Chiecca G., Scardoni A., Minerva M., Mantecca R., Musarò P. (2021). Application of the “immunization islands” model to improve quality, efficiency and safety of a COVID-19 mass vaccination site. Ann. Ig..

[35] Kamolratanakul S., Pitisuttithum P. (2021). Human Papillomavirus Vaccine Efficacy and Effectiveness against Cancer. Vaccines.

[36] Signorelli C., Odone A., Ciorba V., Cella P., Audisio R.A., Lombardi A., Mariani L., Mennini F.S., Pecorelli S., Rezza G. (2017). Human papillomavirus 9-valent vaccine for cancer prevention: A systematic review of the available evidence. Epidemiol. Infect..

